# Post-Exposure Anti-Ricin Treatment Protects Swine against Lethal Systemic and Pulmonary Exposures

**DOI:** 10.3390/toxins12060354

**Published:** 2020-05-28

**Authors:** Reut Falach, Anita Sapoznikov, Yentl Evgy, Moshe Aftalion, Arik Makovitzki, Avi Agami, Avishai Mimran, Elad Lerer, Alon Ben David, Ran Zichel, Shahaf Katalan, Amir Rosner, Tamar Sabo, Chanoch Kronman, Yoav Gal

**Affiliations:** 1 Department of Biochemistry and Molecular Genetics, Israel Institute for Biological Research, Ness-Ziona 76100, Israel; reutf@iibr.gov.il (R.F.); anitas@iibr.gov.il (A.S.); yentle@iibr.gov.il (Y.E.); moshea@iibr.gov.il (M.A.); tamars@iibr.gov.il (T.S.); 2 Department of Biotechnology, Israel Institute for Biological Research, Ness-Ziona 76100, Israel; arikm@iibr.gov.il (A.M.); avia@iibr.gov.il (A.A.); avishaim@iibr.gov.il (A.M.); eladl@iibr.gov.il (E.L.); alonb@iibr.gov.il (A.B.D.); ranz@iibr.gov.il (R.Z.); 3 Department of Pharmacology, Israel Institute for Biological Research, Ness-Ziona 76100, Israel; Shahafk@iibr.gov.il; 4 Veterinary Center for Preclinical Research, Israel Institute for Biological Research, Ness-Ziona 76100, Israel; amirr@iibr.gov.il

**Keywords:** ricin, antitoxin, swine, neutralizing units, intratracheal, intramuscular

## Abstract

Ricin, a plant-derived toxin originating from the seeds of *Ricinus communis* (castor bean plant), is one of the most lethal toxins known. To date, there is no approved post-exposure therapy for ricin exposures. This work demonstrates for the first time the therapeutic efficacy of equine-derived anti-ricin F(ab’)_2_ antibodies against lethal pulmonary and systemic ricin exposures in swine. While administration of the antitoxin at 18 h post-exposure protected more than 80% of both intratracheally and intramuscularly ricin-intoxicated swine, treatment at 24 h post-exposure protected 58% of the intramuscular-exposed swine, as opposed to 26% of the intratracheally exposed animals. Quantitation of the anti-ricin neutralizing units in the anti-toxin preparations confirmed that the disparate protection conferred to swine subjected to the two routes of exposure stems from variance between the two models. Furthermore, dose response experiments showed that approximately 3 times lesser amounts of antibody are needed for high-level protection of the intramuscularly compared to the intratracheally intoxicated swine. This study, which demonstrates the high-level post-exposure efficacy of anti-ricin antitoxin at clinically relevant time-points in a large animal model, can serve as the basis for the formulation of post-exposure countermeasures against ricin poisoning in humans.

## 1. Introduction

Ricin is a member of the type II ribosome-inactivating protein family and as such, consists of two polypeptide chains (A and B) linked by a disulfide bond. The B chain is a lectin, which binds to galactose residues on the cell surface. The A chain possesses RNA N-glycosidase activity that irreversibly inactivates the 28S rRNA of the mammalian 60S ribosome subunit, subsequently arresting cell protein synthesis [1]. Ricin is classified as a Category B agent by the US Center for Disease Control and Prevention (CDC) and is considered a potential bioterror agent due to its high availability and ease of preparation [2]. In fact, a recent appraisal of potential bioweapon threats by a wide panel of biodefense experts ranked ricin as the topmost of 33 potential biothreat agents, surpassing smallpox, botulinum toxin and hemorrhagic viruses [3]. 

Ricin toxicity depends largely on the route of exposure, with inhalational and parenteral exposures being highly fatal [4]. Currently, passive immunization with anti-ricin neutralizing antibodies is the only post-exposure treatment found to be effective against ricin intoxications in pre-clinical settings [5,6,7]. 

In the past decade, our group focused on pathogenesis and anti-ricin antibody-based protection studies in a small animal model, the mouse [8,9,10,11,12,13,14]. However, a large animal was required in order to establish a model that is more relevant to disease progression and post-exposure protection in humans. Due to the high resemblance between the human and porcine cardiovascular and pulmonary systems [15,16], swine were chosen for the study of ricin pathogenesis and post-exposure treatment efficacy. Indeed, the swine animal model served us in the past to determine that pulmonary exposure to ricin leads to the development of bona fide acute respiratory distress syndrome [17]. Unlike with the mouse model, protection experiments aiming to assess antibody-based post-exposure efficacy in swine, require considerably large amounts of antitoxin, and for this purpose, we recently produced F(ab’)_2_-based anti-ricin antitoxin from hyperimmune plasma harvested from a ricin-vaccinated horse [14].

In the present study, we examined the therapeutic efficacy of equine-derived anti-ricin antitoxin following exposure of swine to a lethal dose of ricin by either the pulmonary (intratracheal) or systemic (intramuscular) route of exposure. To date, there is no definition of anti-ricin neutralizing units, so it was essential to develop a method for quantifying the anti-ricin neutralizing units (NU) in the different batches employed in these studies. Although methods for ascertaining the protectivity of anti-ricin antibodies may be found in the literature, these tests are based on in vitro assays, and are not good correlates for in vivo neutralization of ricin. We therefore developed an in vitro/in vivo assay for determining anti-ricin NUs, based on a previously established method for NU quantification of anti-botulinum and anti-snake venom antitoxins [18,19]. This method enabled us to compare the protective capabilities of our horse-derived antitoxins following intramuscular (i.m.) and intratracheal (i.t.) ricin intoxications in swine and to determine that the window of opportunity for effective protection against systemic ricin exposures is significantly wider than that against pulmonary exposures. 

## 2. Results

### 2.1. Intramuscular Ricin Toxicity Determination in Pigs

In a previous study, we determined that intratracheal application of ricin to swine, at a dose of 3 µg per kilogram body weight, leads to the death of all the animals within 30–70 h post-exposure [17]. To assess and compare the post-exposure efficacy of anti-ricin antibodies in swine exposed to ricin by pulmonary (intratracheal) and systemic (intramuscular) exposure routes, we first determined the i.m.-administered ricin dose that brings about death of all intoxicated animals within a similar timeframe. To this end, swine were injected into the left thigh muscle with various doses of ricin within the range of 2.5–20 µg ricin per kilogram body weight (up-down procedure) and then monitored for 14 days (Table 1). Application of ricin at doses of 10 or 20 µg per kilogram body weight resulted in death within 24 h, whereas administration of ricin at the low dose of 2.5 µg/kg did not entail fatal consequences. Intermediate doses of 5–6 µg/kg led to the death of all animals; however, the time to death of individual animals varied strikingly, and in some cases, death occurred way beyond 70 h. In contrast, toxin administration at a dose of 7.5 µg/kg led to the death of the 3 pigs tested, within 45–48 h post-exposure, and therefore, this ricin dose was employed in subsequent experiments. All experiments in which antibody therapy was tested included a control pig that was exposed to ricin and not treated with antibodies. Death of the control pig consistently occurred within the time range of 30–70 h post-exposure.

Swine intoxicated with ricin at 7.5 µg/kg developed signs of intoxication, including lethargy, reduced motor activity and abstinence from food. Cyanosis of body extremities was observed a few hours before death in most animals. 

### 2.2. Antitoxin Treatment against Intratracheal or Intramuscular Intoxications in Swine

**Figure 1:**
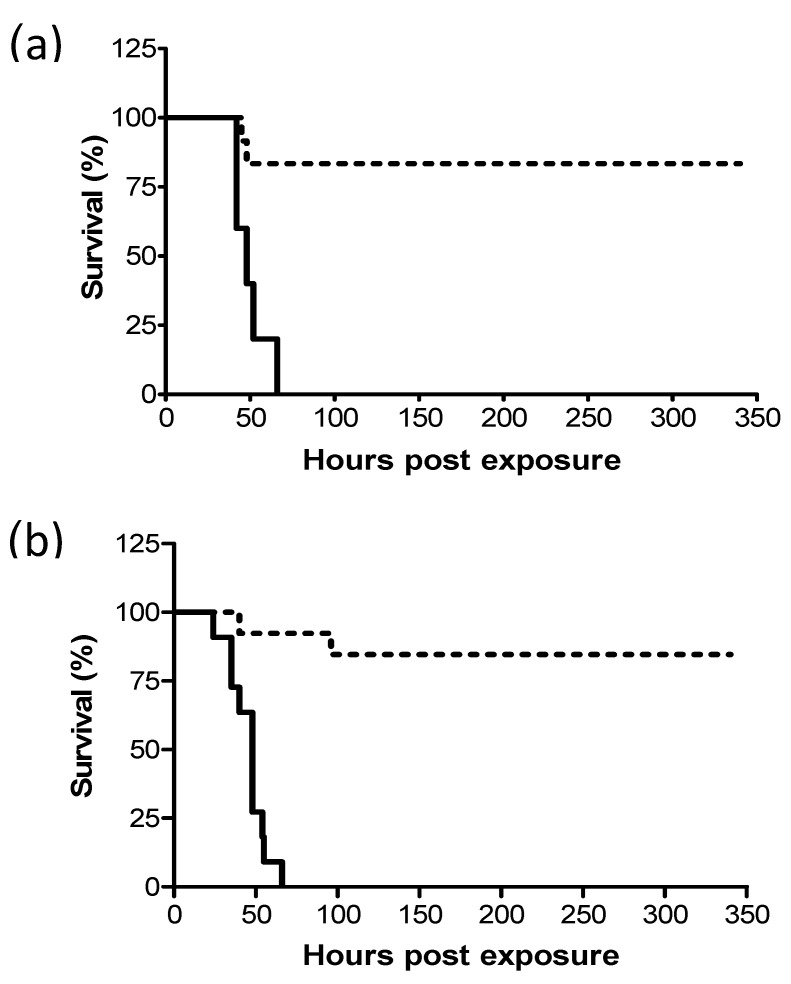
Kaplan–Meier curves of ricin-intoxicated swine that were subjected to treatment with anti-ricin equine-derived F(ab’)_2_ at 18 h post-exposure. Swine exposed to a lethal dose of ricin were treated intravenously (i.v.) or not with anti-ricin F(ab’)_2_ at a dose of 3.5 mL/kg body weight 18 h later and then monitored for survival up to 14 days. (**a**) intramuscular (i.m.)-exposed swine: solid line, no Ab treatment (0/5 live/total), dashed line, Ab-treated (10/12 live/total, 83% survival). (**b**) intratracheal (i.t.)-exposed swine: solid line, no Ab treatment (0/11 live/total), dashed line, Ab-treated (11/13 live/total, 85% survival).

Previous studies have shown that following intranasal instillation of a lethal dose of ricin, mice can be protected by treatment with equine-derived anti-ricin antitoxin [14] and that survival rates correlated inversely to the lapse of time between exposure and treatment. In this work, we examined whether pulmonary (i.t.) and systemic (i.m.) ricin intoxications in the swine model could be similarly alleviated by antibody treatment. To this end, ricin at lethal doses of 7.5 and 3 µg/kg body weight were administered i.m. and i.t. respectively, to groups of swine. Eighteen hours later, the swine were intravenously administered equine-derived antitoxin at a volume of 3.5 mL/kg body weight and monitored for survival up to 14 days following exposure. This treatment with equine-derived anti-ricin antitoxin was found to confer nearly equal and high-level protection of 83% and 85% to the i.m.- and i.t.-exposed swine, respectively (Figure 1).

### 2.3. Antitoxin Neutralizing Unit Determination and Quantification

Due to the large volume of equine anti-ricin F(ab’)_2_ antitoxin needed for experiments in the swine model, the protection experiments in pigs exposed via the pulmonary and systemic routes were performed with two different batches of the F(ab’)_2_ antitoxin: RR-001 and RR-002, respectively. As such, one cannot compare, in a meaningful manner, the protective potential of anti-ricin antibodies against pulmonary and systemic exposures, unless the anti-ricin neutralizing units in each of the antitoxin preparations is determined. We therefore set out to establish a robust and reproducible assay for anti-ricin neutralizing antibodies, based on the in vitro/in vivo European pharmacopeia assay for anti-botulinum neutralizing antibodies [18] and the World Health Organization (WHO) guidelines for the production, control and regulation of snake antivenom immunoglobulins assays [19]. First, in the in vitro stage, a fixed amount of ricin is allowed to interact with a set of dilutions of the tested anti-ricin preparation. The following in vivo step involves i.m. administration of these mixtures to groups of mice, which are then monitored for survival over a period of 14 days. The survival rates in the different groups of mice are a reflection of the amounts of residual non-neutralized toxin in each of the toxin/antibody mixtures.

The median effective dose (ED_50_) and neutralizing unit contents (NU/mL = 1000/ED_50_) of each anti-ricin batch were assessed in three independent in vitro/in vivo experiments, each experiment comprising 5 groups of mice with 4 mice per group (Table 2). The coefficient of variation (%CV) values of the ED_50_ measured for RR-001 and RR-002, ~10% and ~20% respectively, reflect differences in survival rates which occurred in only 1 to 2 of the 5 mice groups in each experiment. As described in the Methods Section, each experiment included, alongside with the samples tested, a standard anti-ricin sample which was examined at different dilutions and served as an internal control.

Assessment of the two antitoxin preparations employed in the swine intoxication/treatment experiments, RR-001 and RR-002, by the in vitro/in vivo assay (Table 2) allowed us to determine their neutralizing unit contents, 1391 ± 121 and 1567 ± 257 NU/mL, respectively.

It therefore follows that the high-level protection (>80%) of the pulmonary and systemic exposed swine was achieved by administration of similar doses of anti-ricin antitoxin, 4869 and 5485 NU per kilogram body, respectively.

### 2.4. Anti-Ricin Antibody Dose Requirement for Treatment—Comparison between Pulmonary and Systemic Ricin Intoxications

We next determined the minimal protective dose in pigs after pulmonary or systemic ricin exposures. Decreasing antitoxin doses were administered to i.t. and i.m. ricin-intoxicated swine at 18 h post-exposure, the time point at which high survival rates (>80%) were reached following application of anti-ricin antibodies at a dose of ~5000 NU/kg body weight. In the case of i.m.-intoxicated swine, decreasing the therapeutic dose from 5500 to ~1600 NUs per kilogram body weight did not reduce survival rates in a significant manner (Table 3). Surprisingly, an additional small reduction in the therapeutic dose, to ~1200 NUs per kilogram body weight, significantly compromised survival, and only 47% survival was observed. In comparison to i.m.-intoxicated swine, pulmonary intoxicated swine required higher doses of the anti-ricin antibodies for achieving high-level survival. Reducing the therapeutic dose applied to i.t.-intoxicated swine, from ~5000 to ~2100 NUs per kilogram body weight, resulted in the plummeting of survival rates from 85% to 29%. Interestingly, with the exception of a single animal (i.t. exposure, treatment with 3.5 mL/kg, time-to-death = 90 h), treatment with anti-ricin anti-toxin, following ricin intoxication by either route of exposure and at various treatment dosages, did not significantly extend the time-to-death of the animals which succumbed.

**Figure 2:**
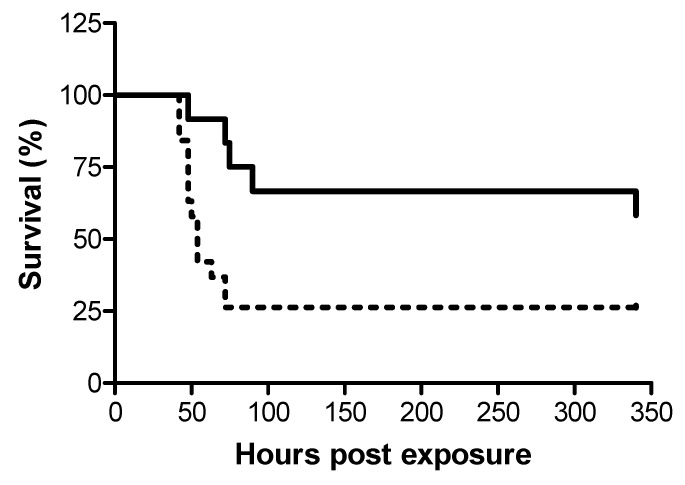
Kaplan–Meier curves of ricin-intoxicated swine that were subjected to treatment with anti-ricin equine-derived F(ab’)_2_ at 24 h post-exposure. Swine exposed to a lethal dose of ricin were treated i.v. with anti-ricin F(ab’)_2_ at a dose of ~5000 NU/ kg body weight 24 h later and then monitored for survival up to 14 days. Solid line, i.m.-intoxicated swine (7/12 live/total = 58% survival), dashed line, i.t.-intoxicated swine (5/19 live/total = 26% survival).

In view of the dissimilar dosages required for efficient treatment following i.m. and i.t. exposures to ricin, we examined whether disparate protection efficacy is manifested also in terms of the window of opportunity for effective treatment against the two modes of exposure. To this end, ricin at doses of 7.5 and 3 µg/kg body weight were administered i.m. and i.t. respectively, to groups of swine, and 24 h later, the swine were intravenously administered equine-derived antitoxin at the dose which provided high-level and equal protection at 18 h post-exposure, ~5000 NU (3.5 mL/kg body weight). While treatment with antitoxin at this dosage at 24 h post-exposure conferred protection to 58% of the i.m.-exposed swine, only 26% of the i.t.-exposed swine were rescued (Figure 2).

## 3. Discussion

Although prophylactic anti-ricin vaccines are being developed [20], the only post-exposure measure found to be effective against ricin intoxications in pre-clinical settings is passive immunization with anti-ricin neutralizing antibodies [21,22,23]. In a recent study, ovine-derived anti-ricin antitoxin was shown to confer high-level protection at late time-points (24–30 h post-exposure) following exposure to aerosolized ricin, however these studies were confined to a small animal model, the mouse [24]. In another study, monoclonal anti-ricin antibodies were shown to protect nonhuman primates; however, high-level protection was reached only when treatment was given at 4 h post-exposure to a lethal dose of aerosolized ricin. When treatment was postponed to 12 h post-exposure, protection levels were as low as 20% [25]. In the present study, to facilitate significant evaluation of anti-ricin protectivity in high numbers of a large animal model, we evaluated the therapeutic potential of equine-derived anti-ricin F(ab’)_2_ in swine.

Appraisal of the efficacy of an anti-ricin antitoxin requires a reliable, reproducible and accurate antibody NU quantification assay. Since in-vitro assays, such as those based on ricin-induced protein synthesis inhibition, do not reiterate the multifaceted pathologies observed in vivo following ricin intoxication (i.e., inflammation, vasculopathy), the ability of an antitoxin preparation to neutralize ricin in cell-based assays may not reflect its protective potential in-vivo. We therefore established an anti-ricin NU assay which is based on an in vitro/in vivo setting. In this assay, a challenge dose of ricin is first incubated in-vitro with different volumes of the tested anti-ricin antitoxin, the ricin/antibody mixtures are then injected to different groups of mice and anti-ricin NU quantitation is based on the antibody’s ability to confer protection to the animals. Similar assays have been employed by the European pharmacopeia and the WHO for assessing neutralizing units in preparations of botulinum and antivenom antitoxins, respectively [18,19].

The anti-ricin F(ab’)_2_ antibodies that served for post-exposure protection of intoxicated swine were derived from hyperimmune plasma, harvested from a horse that was vaccinated with monomerized inactive ricin [14]. Typically, repeated large-scale plasmapheresis at 3-month intervals provides an annual yield of ~40 L of hyperimmune plasma, which are then converted to ~4 L of anti-ricin F(ab’)_2_. Employing the anti-ricin NU quantification assay described above, the two F(ab’)_2_ batches used in the current study, RR-001 and RR-002, were determined to contain 1390 and 1560 NUs/mL, respectively. Treatment at 18 h after exposure with different doses of anti-ricin antibodies allowed us to determine that approximately 1600 and 5000 NUs per kilogram body weight provide high-level protection to i.m.- and i.t.-exposed swine, respectively. Taking into account that toxin doses applied in i.m. and i.t. exposures were 7.5 and 3 microgram per kilogram body weight respectively, a stoichiometric equivalent of ~8 times more anti-ricin antibodies were required for effective protection against pulmonary (i.t.) exposure to the toxin. The requirement for greater amounts of antibody in the case of pulmonary exposure may stem from the fact that in this case, nearly all of the toxin remains confined within the lungs [26] and therefore, effective treatment requires that a sufficient amount of intravenously applied antibodies find their way to this organ. In contrast, ricin toxin applied by systemic exposure is expected to be more accessible to intravenously administered antibodies. Notably, experiments carried out recently in our laboratory (data not shown) imply that systemic exposure to ricin via intramuscular application leads to a severe vasculature disorder, in which case, the intravenously administered antibodies are expected to be in intimate proximity to the site of ricin activity.

Assessment of anti-ricin F(ab’)_2_ antibody efficacy at 24 h post-exposure demonstrated that at late time points, significantly higher levels of protection can be conferred to systemically exposed swine, as compared to pulmonary-exposed animals (see Figure 2). A limitation to the present study is that protection efficacy at this time point was probed only with a single dose of antitoxin (~5000 NU/kg body weight), that which provided high-level protection against both i.m. and i.t. intoxications at 18 h post-exposure. Further studies are needed to determine whether higher doses of anti-ricin F(ab’)_2_ antibody will provide better protection as late as 24 h post-exposure.

## 4. Conclusions

In the current study, we presented for the first time a highly efficient post-exposure treatment for both systemic and pulmonary ricin intoxications (i.t. and i.m.) in a large animal model at clinically relevant time points. We further demonstrated that the anti-ricin antibody dose needed for effective protection following systemic exposure is considerably lower than that needed for dealing with pulmonary intoxication. Based on the minimal therapeutic doses that conferred high-level post-exposure protection to the swine and pending a linear extrapolation from swine to humans, doses of 82,000 and 340,000 NUs would be required for high-level protection of average human adults (70 kg) that have been exposed to a lethal dose of ricin via parenteral or inhalational routes of exposure, respectively.

## 5. Materials and Methods

### 5.1. Ricin Preparation

Crude ricin was prepared from seeds of endemic *R. communis*, essentially as described previously [8]. Briefly, seeds were homogenized in 5% acetic acid/phosphate buffer (Na_2_HPO_4_, pH7.4), the homogenate was centrifuged and the clarified supernatant containing the toxin was subjected to ammonium sulfate precipitation (60% saturation). The precipitate was dissolved in phosphate buffered-saline (PBS) and dialyzed extensively against the same buffer. The toxin preparation appeared on a Coomassie Blue-stained non-reducing 10% polyacrylamide gel as 2 major bands of molecular weight, approximately 65 kDa (= ricin toxin, ~80%) and 120 kDa (= ricinus communis agglutinin (RCA), ~20%). Protein concentration was determined by 280 nm absorption in a Nanodrop device (Thermo Fisher Scientific, Waltham, MA, USA).

### 5.2. Antitoxin Preparation

Generation of monomeric non-toxic ricin antigen and horse immunization procedures were described previously [14]. Plasmapheresis was conducted once in three months utilizing a veterinary plasmapheresis instrument (plasma collection system, PCS-2, Haemonetics Corporation, Braintree, MA, USA) and hyperimmune plasma were stored at −20 °C until further use. Anti-ricin F(ab’)_2_ preparations were generated by pepsin (1200 U/mL pepsin A from porcine stomach mucosa, Sigma, Steinhaim, Germany) cleavage of the Fc fragments, purification of the F(ab’)_2_ fragments and concentration of the antitoxin solution.

### 5.3. Animals

Animal experiments were performed in accordance with the Israeli law and were approved by the Ethics Committee for Animal Experiments at the Israel Institute for Biological Research (Protocol # P-11-2016, 3.11.2013; #P-08-2017, 28.9.2017; P-02-2018, 21.3.2018; P-08-2018, 24.6.2018). Treatment of animals was in accordance with regulations outlined in the United States Department of Agriculture (USDA) Animal Welfare Act and the conditions specified in the Guide for Care and Use of Laboratory Animals (National Institute of Health, 1996).

Female CD-1 mice (Charles River Laboratories Ltd., Canterbury, UK), weighing 27–32 g, were used for anti-ricin neutralizing unit determination. Prior to all studies, the animals were habituated to the experimental animal unit for at least 5 days. All mice were housed in filter-top cages in an environmentally controlled room and maintained at 21 ± 2 °C and 55% ± 10% humidity. Lighting was set to mimic a 12/12 h dawn to dusk cycle. Animals had access to food and water ad libitum.

Young female pigs (Topigs 20, a cross between Yorkshire female and Landrace male, 12–21 kg, age 12–18 weeks) were obtained from an approved commercial source (Van Beek, Lelystad, Netherlands), fed on standard pig diet and housed in a purpose-built animal holding facility for 4–8 days prior to the start of the experiment. Animals were allowed ad libitum access to food. 12 h before the experimental procedure, food was withdrawn, while water remained freely available. Lighting was set to mimic a 12/12 h dawn to dusk cycle.

### 5.4. Median Effective Dose (ED_50_) Assay for Measuring Anti-Ricin Neutralizing Antibodies—In Vitro/In Vivo Assay in Mice

Establishment of this assay for measurement of the capability of an anti-ricin antitoxin to neutralize the lethal effect of ricin first required the determination of the lethal potency of the toxin. Employing the median (50%) lethal dose (LD_50_) assay, the median lethal dose of the crude ricin preparation in intramuscularly exposed mice (=1 mouse i.m. LD_50_) was determined to be 13 µg /kg body weight.

The ricin “challenge dose” in the in vitro/in vivo assay was set to be 24_i.m._ LD_50_/mouse (312 µg/kg), this high dose stemming from the requirement that at least one of the set of diluted antibody/toxin mixtures will contain non-neutralized toxin equivalent to 2–4 LD_50_, thereby leading to the death of all members of the mice group to which this antibody/toxin mixture was applied.

In the in vitro stage, 5 dilutions of the tested anti-ricin preparation (dilution factor = 1.2, adjusted to a constant final volume with PBS) were mixed with the ricin “challenge dose” and incubated for 1 h at 37 °C. Following the European pharmacopeia guidelines for determining anti-botulinum neutralizing antibodies, the range of antibody dilutions included an antibody/toxin mixture which supports full protection of at least one mouse group and an antibody/toxin mixture which brings about full death of at least one mouse group.

In the in vivo stage of the assay, aliquots of each of these antibody/toxin mixtures were injected i.m. to the hind limb of mice (5 groups of mice, 4 mice per group) and mortality was monitored over 14 days. The ED_50_ dose, analyzed by the Spearman–Karber procedure [27], is defined as the volume of anti-ricin (µl) calculated to confer survival to 50% of the mice when applied in an antitoxin/24_i.m._ LD_50_ ricin mixture. In all in vitro/in vivo assays, a set of dilutions of a designated hyper-immune anti-ricin plasma co-incubated with the ricin “challenge dose” served as an internal control standard and was injected to 4 groups of mice (4 mice per group). A test was considered valid only if at least one group of mice was fully protected, and one entire group of mice died following application of the internal control standard/ricin mixtures.

### 5.5. Ricin Exposure and Antibody Treatment in Swine

Pulmonary exposure: swine were intratracheally exposed to ricin as described previously [17]. Briefly, anesthetized swine were intubated with a cuffed endotracheal tube and intratracheal penetration was verified by capnography and chest X-ray. Crude ricin (3 µg/mL/kg) was instilled in two portions while tilting the raised supine animal (~30°) from right to left [17].

Systemic exposure: swine were exposed to a lethal dose of crude ricin (7.5 µg/kg) administered by an intramuscular injection to the left thigh muscle (10 µL/kg).

Post-exposure treatment: equine-derived anti-ricin F(ab’)_2_ antitoxin was administered intravenously to the ear vein of the ricin-intoxicated swine at the indicated doses and time-points. Swine were monitored for survival up to 14 days following exposure.

## Figures and Tables

**Table 1:** Determination of ricin lethal dose in intramuscular (i.m.)-exposed swine.

Pig ID	Weight (kg)	Ricin (µg/kg)	TTD ^a^ (Hours)	MTTD ^b^ ± SD (Hours)
6936	20.1	20	20	22 ± 2.8
7055	20.8	10	24	
7047	14.6	2.5	survived	
6843	14.0	5	46	92 ± 68
9101	17.3	5	90	
9307	14.3	6	53	
9243	13.6	6	53	
9244	14.6	6	190	
6914	14.2	6	210	
6954	13.4	6	47	
6842	12.4	6	47	
7128	14.7	7.5	48	47 ± 2
9317	17.2	7.5	47	
9507	14.9	7.5	45	

^a^ TTD, Time to Death. ^b^ MTTD, Mean Time to Death. SD, standard deviation.

**Table 2:** Antitoxin neutralizing unit quantification.

		RR-001 ^a^			RR-002 ^a^		
		Experiment I	Experiment II	Experiment III	Experiment I	Experiment II	Experiment III
µL/mouse ^b^	1.3	4/4	4/4	nd	nd	nd	nd
	1.08	4/4	4/4	4/4	4/4	4/4	4/4
	0.9	4/4	4/4	4/4	4/4	4/4	4/4
	0.75	4/4	4/4	1/4	4/4	1/4	4/4
	0.63	0/4	0/4	0/4	3/4	0/4	4/4
	0.53	nd	nd	0/4	0/4	0/4	0/4
ED_50_^c^ (µL)		0.68	0.69	0.8	0.6	0.79	0.57
NU/mL		1461	1461	1251	1675	1274	1753
Average NU ^d^/mL ± STDEV		1391 ± 121			1567 ± 257		

^a^ Antitoxin batch. ^b^ Results are presented as number of surviving mice/total mice per group. nd: not done. ^c^ ED_50_: median (50%) effective dose. ^d^ NU: neutralizing unit.

**Table 3:** Anti-ricin antibody dose requirement for treatment in ricin-intoxicated pigs.

Exposure Route/Antitoxin Batch	Treatment Dose		Survival (%) (Survivors/Total)	Time-to-Death (Time Range in Hours *)
	mL/kg	NU/kg		
Intramuscular/RR-002	3.5	5484	83 (10/12)	45, 48
	1.5	2350	80 (12/15)	42, 48, 48
	1	1567	73 (11/15)	42–45
	0.75	1175	47 (7/15)	42–66
	0	0	0 (0/8)	42–60
Intratracheal/RR-001	3.5	4868	85 (11/13)	40, 90
	1.5	2086	29 (4/14)	40–65
	0	0	0 (0/14)	40–60

* hours-to-death values are presented per animal when *n* ≤ 3.
